# Dietary total antioxidant capacity in relation to disease severity and risk of mortality in cirrhosis; results from a cohort study

**DOI:** 10.1016/j.heliyon.2024.e37733

**Published:** 2024-09-12

**Authors:** Fereshteh Pashayee-Khamene, Melika Hajimohammadebrahim-Ketabforoush, Zeinab Heidari, Zahra Yari, Sara Karimi, Mahdi Saber-firoozi, Behzad Hatami, Azita Hekmatdoost

**Affiliations:** a Student Research Committee, Ahvaz Jundishapur University of Medical Sciences, Ahvaz, Iran; b Department of Clinical Nutrition and Dietetics, Faculty of Nutrition Sciences and Food Technology, National Nutrition and Food Technology Research Institute, Shahid Beheshti University of Medical Sciences, Tehran, Iran; c Department of Nutrition Research, National Nutrition and Food Technology Research Institute, Faculty of Nutrition Sciences and Food Technology, Shahid Beheshti University of Medical Sciences, Tehran, Iran; d Liver and Pancreato-biliary Disease Research Center, Digestive Disease Research Institute, Shariati Hospital, Tehran University of Medical Sciences, Tehran, Iran; e Gastroenterology and Liver Diseases Research Center, Research Institute for Gastroenterology and Liver Diseases, Shahid Beheshti University of Medical Sciences, Tehran, Iran

**Keywords:** Liver cirrhosis, Total antioxidant capacity, TAC, ORAC, Severity, Mortality

## Abstract

Liver Cirrhosis, defined as the final stage of chronic liver disease, may become more prevalent in the lower level of body defense against oxidation and inflammation. Therefore, we assessed the association of dietary total antioxidant capacity (DTAC) with the severity and mortality of cirrhosis in a cohort study. 120 newly diagnosed cirrhosis patients from Tehran, Iran, participated in this study. The patients’ habitual diet was assessed using a 168-item validated food frequency questionnaire. Both ferric-reducing antioxidant potential (FRAP) and oxygen radical scavenging capacity (ORAC) methods were computed to achieve DTAC scores. The association between DTAC with disease severity and mortality was estimated by multivariate linear regression and cox proportional hazards regression models. Dietary total antioxidant capacity-ORAC had a significant inverse association with disease severity in both crude and adjusted models (*P* for trend: <0.001 and 0.016 respectively). The risk of mortality in the first and second tertiles of ORAC was 5.56 (95 % CI: 2.25–13.75; P = 0.002) and 3.20 (95 % CI: 1.25–8.19; P = 0.015) higher than those in the third category, respectively. In conclusion, a higher antioxidant capacity of diet is associated with less disease severity and mortality risk in cirrhosis.

## 1 Introduction

Liver cirrhosis is a serious liver condition characterized by the death of liver cells and the formation of scar tissue in the liver. It is considered the final stage of liver disease and is a significant public health concern due to its high mortality rate [1]. Chronic liver diseases become more prevalent when the body's defenses against oxidation and inflammation are depleted [2]. Pathways associated with oxidative stress and inflammation can affect the cellular and tissue processes that play a role in the formation and progression of liver fibrosis [3,4]. When there is an excessive buildup of free fatty acids in the liver, the liver cells increase their breakdown of fatty acids, resulting in the production of reactive oxygen and nitrogen species by various organelles. These reactive species can harm liver cells directly by damaging their molecules and indirectly by causing inflammation [5]. The accumulation of these reactive species can deplete the liver's antioxidants, leading to liver damage and cell death through different mechanisms, including the activation of hepatic stellate cells and Kupffer cells, depletion of ATP, and liver fibrogenesis [6]. Inflammation is a crucial part of the immune response, and continued exposure to inflammatory triggers and oxidative stress may result in cellular damage, lipid accumulation, and fibrosis [3,4]. Studies have shown that diet can play a role in modulating inflammation and oxidative stress [7,8].

At present, the total antioxidant capacity of the diet (DTAC) is considered a valuable measure for assessing the overall antioxidant capacity of foods [9]. In this particular context, the total antioxidant capacity of foods has been suggested as a means of examining the positive effects on health that can result from consuming mixed diets that contain antioxidants [[10], [11], [12]]. DTAC is preferred over a simple sum of individual dietary antioxidants as it reflects the combined capacity of all dietary antioxidants [9]. This capacity refers to the ability of dietary antioxidants to remove performed free radicals [12]. Numerous studies suggest that higher DTAC is linked with a reduced risk of adverse health outcomes, including cardiovascular diseases, type 2 diabetes, cancer-related deaths, and obesity [[13], [14], [15], [16]]. Previous research on non-alcoholic steatohepatitis indicated a reverse correlation between DTAC and hepatic injury [17]. However, to date, there have been no reports in the existing literature on the examination of DTAC in outpatients with cirrhosis. Therefore, the goal of this current study is to explore the potential correlation between DTAC and cirrhosis severity, Survival time, and mortality in cirrhosis patients.

## 2 Methods

### 2.1 Study design and subjects

One hundred twenty patients, 20–70 years old, with at least six months of cirrhosis diagnosis, were enrolled in this cohort study between December 2016 and September 2018 in Iran. We included patients from all various etiology which can lead to liver cirrhosis finally, such as viral infections, nonalcoholic steatohepatitis, alcoholic liver diseases, autoimmune disorders, and cryptogenic cirrhosis. Cirrhosis was diagnosed based on clinical data and imaging techniques (ultrasound and, or computed tomography). Patients were asked to fill out a questionnaire with demographic characteristics such as age, sex, smoking status, disease history, and a food frequency questionnaire (FFQ) at the beginning of the study. Pregnant women and patients with pancreatitis, cancer, or other chronic diseases were excluded from this study. This study was conducted in accordance with the ethical guidelines of the Helsinki Declaration. The written informed consent form was signed and dated by all participants. This ethics committee of the National Nutrition and Food Technology Research Institute approved the study protocol (ethnical NO: IR. SBMU.NNFTRI.1396.186.). Written Informed consent was obtained from all patients before inclusion.

### 2.2 Data collection

In this study, trained dietitians collected sociodemographic, lifestyle, dietary, and clinical information using questionnaires. Child-Pugh and Model for End-stage Liver Disease (MELD) classifications were used to grade liver disease severity. Scores were calculated based on serum albumin, total bilirubin, international normalized ratio (INR), and the presence of ascites or encephalopathy.

### 2.3 Anthropometric assessment

An anthropometric measurement was performed by a dietitian trained in anthropometry. A digital scale (SECA, Germany) was used to measure weight without shoes, with minimum clothes, and to the nearest 100 g. Using a mounting tape, height was measured in a standing position with no shoes to the nearest 0.5 cm (SECA, Germany). Using a weight (kg) divided by a height in square meters (m^2^), the body mass index (BMI) is calculated.

### 2.4 Dietary assessment

Participants’ dietary intakes were assessed in a face-to-face interview using a validated semi-quantitative food frequency questionnaire with 168 food and beverage items [18]. A manual for household measurements was used to convert intake frequencies into daily grams of food intake [19]. Food composition tables from the USDA were used to calculate energy, nutrients, and flavonoids. Traditional Iranian foods were analyzed based on the Nutrients Composition of Iranian Foods (NCIF), which does not include traditional bread in the USDA database.

### 2.5 TAC calculation

Based on various methods, DTAC estimates the number of free radicals accumulated by dietary antioxidants and the number of free radicals inactivated by them. Based on the Antioxidant Food Table, we calculated FRAP scores that measure a food's ability to reduce ferric ions into ferrous ions in vitro [20]. Also, ORAC can be used to assess the ability of a test sample to inhibit peroxyl radical oxidation by using the USDA database of Oxygen Radical Absorbance Capacity of Selected Foods [21]. The values are expressed as mmol Trolox equivalent (mmol TE/100 gr). In order to calculate the overall DTAC score, the daily intake of each food was multiplied by its FRAP and ORAC values, then the average of these values was added. The DTAC assessment did not include detailed information on vitamin supplementation since such data is unavailable.

### 2.6 Statistical analysis

The data were analyzed using the statistical package IBM SPSS, version 22.0 (Statistical Package for the Social Sciences, IBM Corp., Armonk, New York, USA). The normality of data was analyzed using the Kolmogorov-Smirnov test. The quantitative data with normal distributions are described as mean ± standard deviation (SD). Categorical data were reported as frequencies and percentages. To find the difference between variables based on MELD score's tertiles concerning quantitative and qualitative variables, the one-way ANOVA test and Chi 2 test were used, respectively. Spearman's correlation test was run to find the correlation between categorical and quantitative variables. To assess the relation between dietary antioxidant capacities with disease severity (based on MELD scores) and also potential mortality hazards, multivariate linear regression, and cox proportional hazards regression models were used, respectively, in the stepwise method. Differences in survival time between patients with cirrhosis in the three groups based on dietary total antioxidant capacity (DTAC) were assessed by Kaplan-Meier survival analysis and compared statistically using the log-rank test. According to some similar previous studies [22],age, sex, BMI, total calorie, calcium, protein intake, and ascites were selected as confounder variables to enter regression models. In all analyses, P < 0.05 was considered statistically significant.

## 3 Results

**Fig. 1:**
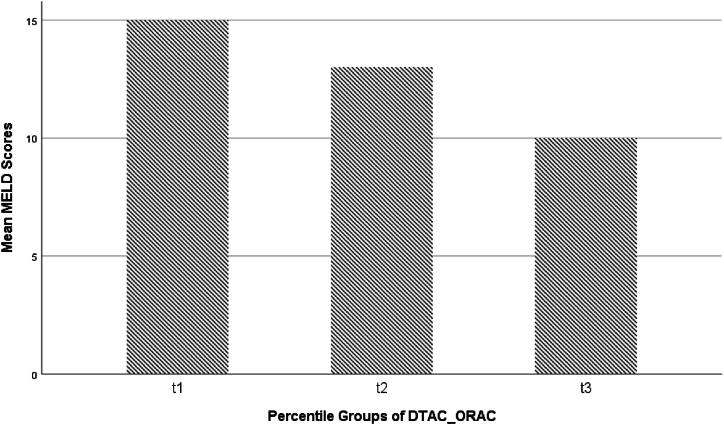
Variation of the MELD scores (mean ± SD) across to different percentile groups based on DTAC-ORAC. t1: first tertile; t2: second tertile; and t3: third tertile.

**Fig. 2:**
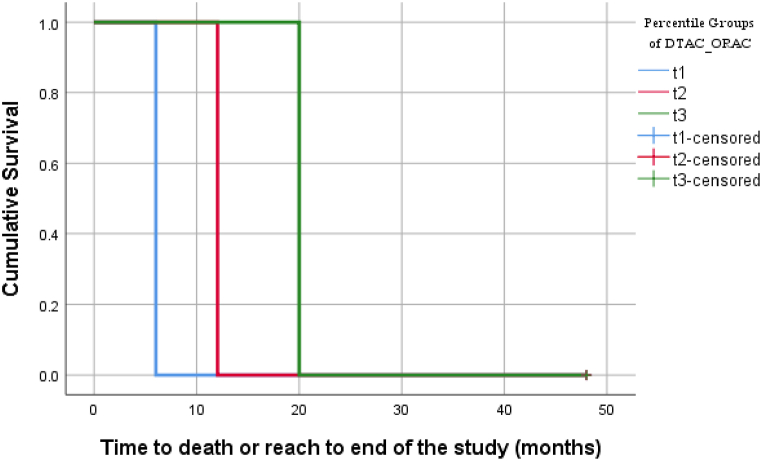
Survival plot which shows the cumulative survival in different tertiles of DTAC-ORAC.

Table 1 demonstrates the clinical characteristics and dietary intakes of patients with cirrhosis based on their disease severity. In our study mean ± SD of MELD scores within groups of Child-Pugh score A, B, and C were 9.77 ± 2.84, 15.03 ± 5.83, and 27.36 ± 8.82, respectively. The result showed a significant positive correlation between MELD and Child-Puph scores (rs, +0.63 P-value <0.001). On the other hand, because of the qualitative nature and unequal categorization of the Child-Pugh score in this study, statistical analyses, for example, multinomial regression, does not have enough validity with uncertain results. Therefore, we reported only MELD in our analyses and tables. Clinical characteristics and nutritional intakes of patients were depicted between different disease severities. Therefore, the MELD score as a representative of disease severity was categorized into tertiles (t1, t2, and t3 from lowest to highest severities, respectively). Ordinal regression has not enough validity and certain result because of the small sample size belonging to each category. Therefore, MELD scores were used as a quantitative dependent variable in the linear regression models. Before this, as Table 1 shows, there were statistically significant differences in the existence of ascites as well as dietary intakes, including total calories, carbohydrates, protein, fat, and calcium between MELD score's tertiles. Results showed that t2 had higher values for total calories (P = 0.004), carbohydrate (P = 0.005) respect to t3. Moreover, t1 had higher values for protein (*P* = 0.007) and total fat (*P* = 0.034) respect to t3. Moreover, dietary total antioxidant capacity (DTAC) based on Oxygen Radical Absorbance Capacity (ORAC) was calculated. Among different food groups, fruits, legumes, and nuts FRAP values differed significantly between MELD score tertiles, with these values being higher in the second tertile of disease severity compared to the other tertiles (Table 1). The post-hoc analysis showed the same pattern results with nutritional intakes as mentioned above. However, regarding the dietary total antioxidant capacity- ORAC, differences between t1 and t3 (*P* = 0.041) and between t2 and t3 (*P* = 0.004) were statistically significant. That's means, the dietary total antioxidant capacity in patients with cirrhosis with the lowest severity was significantly higher than those with the highest severity. In addition, it was more in the medium severity compared to the highest one. The results from multivariate linear regression models showed that there was not any significant relation between the antioxidant capacities of each food group and disease severity. However, dietary total antioxidant capacity- ORAC had an inverse significant association with disease severity in both crude and adjusted models. This happening might be due to the difference of variances that become too small about FRAP of each food group analysis. As Table 2 demonstrates, patients with cirrhosis were located in the first tertile of dietary total antioxidant capacity – ORAC averagely had 5.50 scores of MELD more than the third tertile, as a reference category, in the crude linear regression model (*P* < 0.001). Therefore, their disease had more severity. Moreover, patients with cirrhosis were located in the second tertile of dietary total antioxidant capacity – ORAC averagely had 3.70 scores of MELD more than the third tertile, as a reference category, in the crude linear regression model (*P* = 0.014). After adjusting for age and sex in model 1 then, BMI, total calories, and calcium in model 2, and finally a full-adjust for all confounders in the last model, considering protein intake and ascites at baseline, meaningful of some categories disappeared. It was maybe caused by our small sample size. However, *P* for the trend in any models stayed statistically significant (Table 2, Fig. 1). Results from the Log-Rank test showed that the mean survival time in patients with the lowest intake of DTAC (T1) was significantly lower than the highest one (T3) (P < 0.001) (Table 3, Fig. 2). Moreover, Cox proportional hazards regression model demonstrated that the mortality hazard in T1 and T2 patients was approximately 5.5 and 3.20 compared with T3 patients, respectively (Table 4).

**Table 1:** Clinical characteristics and dietary intakes by tertiles of the MELD score.

	First tertile (t1)	Second tertile (t2)	Third tertile (t3)	*P*-value
MELD ≤9	10 ≤ MELD ≤13	MELD ≥14	(n = 41)	
Parameters	(n = 45)	(n = 34)		
Clinical characteristics				
Women, % (no)	20 (44.4)	9 (26.5)	9 (22)	0.073^*a*^
Age, years	52.42 ± 11.27	54.79 ± 14.19	57.22 ± 10.29	0.177^*b*^
BMI, kg/m2	27.20 ± 5.14	26.70 ± 4.63	25.99 ± 3.91	0.476 ^*b*^
Smoking (yes), n (%)	14 (31.1)	15 (44.1)	18 (43.9)	0.425 ^*a*^
Alcohol consumption (yes), n (%)	7 (15.6)	9 (26.5)	11 (26.8)	0.434 ^*a*^
ALT, mg/dl	42.99 ± 43.09	43.53 ± 45.80	40.40 ± 64.67	0.963 ^*b*^
AST, mg/dl	44.26 ± 33.35	54.68 ± 47.63	66.53 ± 111.57	0.399 ^*b*^
Ascites (yes), n (%)	9 (20)	10 (29.4)	23 (56.1)	0.001 ^*a*^
Dietary intakes				
Energy, kcal	2593.21 ± 1280.15	2905.53 ± 976.61	2075.52 ± 971.71	0.005 ^*b*^
Protein (g/d)	90.16 ± 44.20	109.35 ± 42.54	78.31 ± 42.04	0.009 ^*b*^
Fat (g/d)	78.86 ± 47.98	87.77 ± 38.05	63.41 ± 36.48	0.039 ^*b*^
Carbohydrate (g/d)	405.54 ± 197.26	444.38 ± 160.90	315.81 ± 149.02	0.004 ^*b*^
Saturated fatty acid (g/d)	22.09 ± 15.83	24.14 ± 12.28	18.19 ± 9.42	0.128 ^*b*^
FRAP ^*c*^				
Fruits	4.54 ± 3.40	4.83 ± 3.22	3.18 ± 2.33	0.040 ^*b*^
Vegetables	0.86 ± 0.61	1.04 ± 0.69	0.81 ± 0.73	0.287 ^*b*^
Legumes	0.42 ± 0.34	0.68 ± 0.59	0.40 ± 0.40	0.014 ^*b*^
Grain	1.05 ± 0.82	0.99 ± 0.62	0.99 ± 0.91	0.915 ^*b*^
Nuts	0.34 ± 0.42	0.44 ± 0.61	0.17 ± 0.32 0.036 ^*b*^	
Fat	0.06 ± 0.06	0.07 ± 0.08	0.07 ± 0.13	0.930 ^*b*^
Dairy	0.25 ± 0.28	0.26 ± 0.20	0.18 ± 0.13	0.262 ^*b*^
MFP	0.05 ± 0.04	0.07 ± 0.05	0.05 ± 0.04	0.070 ^*b*^
Coffee	0.35 ± 1.94	0.83 ± 2.68	0.15 ± 0.47	0.290 ^*b*^
Tea	6.21 ± 4.14	6.36 ± 3.98	37 ± 6.20 0.511 ^*b*^	
Other	0.15 ± 0.16	0.38 ± 0.51	0.10 ± 0.16	<0.001 ^*b*^
Micronutrients				
Calcium (mg/d)	1157.69 ± 578.20	1384.91 ± 583.48	980.88 ± 457.50	0.007 ^*b*^
Selenium (mg/d)	118.49 ± 60.89	122.38 ± 54.66	106.25 ± 65.58	0.477 ^*b*^
Vitamin E (mg/d)	15.99 ± 9.28	15.65 ± 6.73	12.27 ± 8.09 0.081 ^*b*^	
Folate (μg/d)	584.40 ± 267.59	704.99 ± 285.23	519.23 ± 200.09	0.007 ^*b*^
Vitamin B12 (μg/d)	2.97 ± 1.86	3.65 ± 2.01	3.04 ± 2.33	0.306 ^*b*^
Vitamin D (μg/d)	1.88 ± 2.16	2.86 ± 2.81	1.52 ± 1.77	0.034 ^*b*^
Vitamin C (mg/d)	192.55 ± 135.93	220.86 ± 121.55	163.35 ± 119.45	0.150 ^*b*^
DTAC_ORAC, mmol TE/100 g	6436.03 ± 14717.51	29376.10 ± 11979.98	19611.96 ± 11336.49	0.004 ^*b*^

^*a*^ Result from Chi-Square test, ^*b*^ One-Way ANOVA test, ^*c*^ Ferric reducing antioxidant power (FRAP), values are expressed in mg μM de FeSO4/g dry basis. Abbreviations: BMI, body mass index; ALT, alanine aminotransferase; AST, aspartate aminotransferase; MFP, meat-fish-poultry; DTAC, dietary total antioxidant capacity; ORAC, oxygen radical absorbance capacity; TE, Trolox equivalents.

**Table 2:** Association between tertiles of dietary total antioxidant capacity – ORAC and cirrhosis severity based on MELD score.

Tertile of dietary total antioxidant capacity – ORAC			*P* for trend
T1 (n = 40)	T2 (n = 40)	T3 (n = 40)	
MELD score, Mean ± SD	15.53 ± 9.78	13.73 ± 5.35 10.03 ± 2.81 0.001^a^	
Crude	5.50 (2.55–8.44)	3.70 (1.01–6.61)	1.00 (Ref.) <0.001
Model 1^a^	6.07 (3.11–9.02)	3.83 (0.88–6.78)	1.00 (Ref.) 0.068
Model 2^b^	4.24 (0.32–8.16)	2.78 (0.57–6.13)	1.00 (Ref.) 0.017
Model 3^c^	4.23 (0.30–8.17)	2.56 (0.87–5.97)	1.00 (Ref.) 0.016

*.Result from one-way ANOVA test.

** Note: Linear regression was used to estimate odds ratios (ORs) and 95 % confidence intervals (CIs) adjusted for multiple covariates in different models.

a Model 1: adjusted for age and sex.

b Model 2: adjusted for model 1 and BMI, total calorie, and calcium.

c Model 3: additionally adjusted for protein intake and ascites at baseline.

**Table 3:** Survival time and disease severity based on tertiles of dietary total antioxidant capacity.

Tertiles of dietary total antioxidant capacity – ORAC				*P*-value
	T1 (n = 40)	T2 (n = 40)	T3 (n = 40)	
Child score, n (%)				019^a^
A	19 (47.5)	21 (52.5)	30 (75)	
B	14 (35)	15 (37.5) 10 (25)		
C	7 (17.5)	4 (10)	0 (0)	
MELD score, n (%)				<0.001^b^
t1	16 (40)	12 (30)	17 (42.5)	
t2	7 (17.5)	7 (17.5)	20 (50)	
t3	17 (42.5)	21 (52.5)	3 (7.5)	
Survival time, months	31.70 (26.61–36.78)	38.22 (34.26–42.18) 46.52 (45.02–48.02)		<0.001^c^

a Result from Fisher's Exact Test.

b Chi-Square Test.

c Log-Rank test.

**Table 4:** Cox proportional hazards regression analysis of mortality based on tertiles of dietary total antioxidant capacity.

β	HR	95 % Confidence Interval			*P*-value
			Lower	Upper	
Tertiles of DTAC a					
T1	1.71	5.56	2.25	13.75	0.002
T2	1.16	3.20	1.25	8.19	0.015

DTAC, dietary total antioxidant capacity; T, tertile.

a Third tertile (T3) was considered as references category. Dependent variable: age, sex, MELD score, ascites, BMI, total calorie, calcium.

## 4 Discussion

The present cohort study investigated the associations between DTAC and mortality among survivors of liver cirrhosis. According to our results, higher dietary total antioxidant capacity was associated with reduced risk of mortality in patients, after full adjustment of confounding factors such as age, sex, MELD score, ascites, BMI, total calorie, and dietary calcium intake. Also, the inverse relationship between DTAC and disease severity was shown in patients. The severity of cirrhosis in the first tertile of DTAC was significantly higher compared to the third tertile. In addition, the results showed that with the increase in the severity of the disease in patients with cirrhosis, food intake decreased, and the prevalence of ascites increased. Moreover, patients with higher DTAC scores showed significantly longer survival times. To the best of our knowledge, the current study is the first prospective study on the association between DTAC and risk of mortality in patients with cirrhosis.

DTAC represents the dietary antioxidant capacity and is considered a good indicator of the quality of the diet (1). DTAC has been reported to be inversely associated with NAFLD (6, 7), which shares similar metabolic abnormalities with cirrhosis. Similar to our findings, accumulating evidence from prior studies have shown that fruits and vegetables consumption, as important sources of antioxidants, is inversely associated with the risk of chronic liver diseases such as cirrhosis [[23], [24], [25], [26], [27]]. Oxidative stress plays a key role in the initiation and progression of liver diseases. Oxidative stress leads to liver inflammation, fibrosis, and cell death by activating redox-sensitive transcription factors and inflammatory mediators [28]. Therefore, antioxidant agents play an imperative role in preventing the initiation and aggravation of liver injury.

Consistent with the findings of the present study, previous studies have shown the relationship between DTAC and the reduction of the risk of mortality [29,30]. Dietary antioxidants, especially when derived from fruits and vegetables, have been reported to be directly associated with reduced risk of chronic diseases [31]. The results of previous studies indicate a direct relationship between DTAC and the consumption of some dietary components and micronutrients containing fiber, folic acid, vitamins A, C, and E, total carotenoids, magnesium, and zinc [32]. A higher DTAC score by strengthening antioxidant defense mechanisms can ultimately prevent liver diseases by preventing lipid peroxidation, and cellular and enzyme damage [33].

In the present study, inverse associations were found between DTAC score and cirrhosis severity. This finding can be due to the protective role of antioxidants against insulin resistance, dyslipidemia, and inflammatory conditions. Dietary antioxidants play a role in reducing lipogenesis and related disorders by inhibiting fat absorption and stimulating fat tissue catabolism [34]. Insulin resistance is also a mediator of the effect of oxidative stress on lipid profile, which has been proven to be inversely related to DTAC (15). Regarding our recent investigations, the role of healthier food choices in cirrhosis severity was concluded that could be due to antioxidant content [35,36].

Another result of the current research was about DTAC and survival time in different severity of cirrhosis. A similar study with which this finding can be compared has not been published. It seems that the possible cause of this relationship is related to the decrease in the severity of cirrhosis with the increase in the DTAC score. Along with this result, malnutrition, as a result of progression in cirrhosis severity, is an independent risk factor for mortality in cirrhosis [37].

Although the present study is the first cohort study to investigate the association between DTAC and mortality risk in patients with cirrhosis, it also had some limitations, including a relatively small study population, inevitable recall bias using food frequency questionnaire (FFQ), and missing of about 15 % of enrolled patients.

## 5 Conclusion

A high DTAC was associated with a reduced risk of mortality in patients with cirrhosis. Also, a higher DTAC score was associated with a decrease in disease severity and an increase in survival time in patients with cirrhosis. Therefore, it can be concluded that consuming a diet with high antioxidant capacity is important in reducing the risk of morbidity and mortality from cirrhosis.

## Data availability statement

Data will be made available on request.

## CRediT authorship contribution statement

**Fereshteh Pashayee-Khamene:** Methodology, Investigation, Formal analysis, Data curation, Conceptualization. **Melika Hajimohammadebrahim-Ketabforoush:** Software, Methodology, Formal analysis. **Zeinab Heidari:** Software, Methodology, Formal analysis, Data curation, Conceptualization. **Zahra Yari:** Writing – original draft, Visualization, Methodology, Investigation. **Sara Karimi:** Investigation, Data curation. **Mahdi Saber-firoozi:** Supervision, Data curation. **Behzad Hatami:** Supervision, Data curation. **Azita Hekmatdoost:** Supervision, Project administration, Methodology, Investigation, Data curation, Conceptualization.

## Declaration of competing interest

The authors declare that they have no known competing financial interests or personal relationships that could have appeared to influence the work reported in this paper.
