# Supplementary material for: Dietary total antioxidant capacity in relation to disease severity and risk of mortality in cirrhosis; results from a cohort study
Source: Heliyon. 2024 Sep 12;10(18):e37733. doi: 10.1016/j.heliyon.2024.e37733 (PMC11417536; doi:10.1016/j.heliyon.2024.e37733)
Supplement: Multimedia component 1 [file mmc1.pdf]

فرم رضايت آگاهانه

۱- اعلام رضايت يا عدم رضايت آزمودني
۲- نام و امضاي آزمودني يا ولي قانوني با ذكر تاريخ
۳- نام و امضاي فردي كه فرم را تحويل مي‌گيرد با ذكر تاريخ

دانشگاه علوم پزشكي و خدمات بهداشتي، درماني شهيد بهشتي

انستيتو تحقيقات تغذيه‌اي و صنايع غذايي كشور

فرم رضايت آگاهانه (الف)

عنوان پروژه: بررسی وضعیت تغذیه ای بیماران مبتلا به سیروز کبدی و ارتباط دریافت های غذایی آنها با پارامترهای بالینی ، بیوشیمیایی، انتروپومتریک و هماتولوژیک در بیمارستان های طالقانی و شریعتی درسال ۹٦

اينجانب با اطلاع كامل از اهداف و روش اجراي پروژه تحقيقاتي فوق‌الذكر و نيز شرايط و نحوه شركت خود در آن، اذعان مي‌دارم كه فرصت كافي براي پرسيدن سئوالات مطروحه و دريافت پاسخهاي مناسب را داشته‌ام، لذا با رضايت كامل، بطور داوطلبانه در اين مطالعه شركت مي‌نمايم.

اين امكان براي من وجود دارد تا در هر زمان كه مايل باشم بدون ارائه دليل از مطالعه خارج شوم و اين موضوع تأثيري بر حقوق قانوني من نخواهد داشت.

نام و نام خانوادگي: ....
تاريخ: ....
امضا: ....

نام و نام خانوادگي تحويل گيرنده فرم:................................

National Nutrition and Food Technology Research Institute

Shahid Beheshti university of medical sciences

Written Consent Form

1. Declaration of consent or dissent by the examinee
2. Name and signature of the examinee or legal guardian along with the date
3. Name and signature of the individual receiving the form along with the date

Project Title: Nutritional assessment in patients with hepatic cirrhosis and its relation with clinical, biochemical, anthropometric and hematologic parameters in Taleghani and Shariati hospitals during winter 2018

I, having full knowledge of the objectives and methods of conducting the aforementioned research project, as well as the conditions and my participation in it, acknowledge that I have had sufficient opportunity to ask questions and receive appropriate answers. Therefore, with complete consent, I voluntarily participate in this study.

I have the opportunity to withdraw from the study at any time without providing a reason, and this will not affect my legal rights.

Name:

Date:

Signature:
