# Supplementary material for: Dietary total antioxidant capacity in relation to disease severity and risk of mortality in cirrhosis; results from a cohort study
Source: Heliyon. 2024 Sep 12;10(18):e37733. doi: 10.1016/j.heliyon.2024.e37733 (PMC11417536; doi:10.1016/j.heliyon.2024.e37733)
Supplement: Multimedia component 2 [file mmc2.pdf]

فرم رضایت آگاهانه

۱- اعلام رضایت یا عدم رضایت آزمودنی

۲- نام و امضاي آزمودني يا ولي قانوني با ذكر تاريخ

۳- نام و امضاي فردي كه فرم را تحويل مي‌گيرد با ذكر تاريخ

دانشگاه علوم پزشكي و خدمات
بهداشتي، درماني شهيد بهشتي

انستيتو تحقيقات تغذيه‌اي و
صنايع غذايي كشور

فرم رضایت آگاهانه (الف)

عنوان پروژه: بررسی وضعیت تغذیه ای بیماران مبتلا به سیروز کبدی و ارتباط دریافت های غذایی آنها با پارامترهای بالینی ،
بیوشیمیایی، انتروپومتریک و هماتولوژیک در بیمارستان های طالقانی و شریعتی درسال ۹۶

اينجانب با اطلاع كامل از اهداف و روش اجراي پروژه تحقيقاتي فوق‌الذكر و نيز شرايط و نحوه شركت خود در آن، اذعان مي‌دارم كه فرصت كافي براي پرسيدن سئوالات مطروحه و دريافت پاسخهاي مناسب را داشته‌ام، لذا با رضايت كامل، بطور داوطلبانه در اين مطالعه شركت مي‌نمايم.

اين امكان براي من وجود دارد تا در هر زمان كه مايل باشم بدون ارائه دليل از مطالعه خارج شوم و اين موضوع تأثيري بر حقوق قانوني من نخواهد داشت.

نام و نام خانوادگي: ..[illegible]..........................

تاريخ: .....۹۶/۱۰/۰..........................

امضا: ....

نام و نام خانوادگي تحويل گيرنده فرم: ....
